# Microbial nitrate removal by waste iron shavings from the biological and catalytic ozonation treated dyeing and finishing wastewater

**DOI:** 10.1186/s13568-016-0309-6

**Published:** 2017-01-03

**Authors:** Jieting Ma, Yunlu Chen, Gang Luo, Jianxin Nie, Zhigang Guo, Yan Liu, Luming Ma

**Affiliations:** 1 Shanghai Key Laboratory of Atmospheric Particle Pollution and Prevention (LAP3), Department of Environmental Science and Engineering, Fudan University, Shanghai, 200433 China; 2 College of Environmental Science and Engineering, Tongji University, Shanghai, 200092 China

**Keywords:** Nitrate removal, Waste iron shavings, Biological and catalytic ozonation treated dyeing and finishing wastewater, Effluent organic matter

## Abstract

The concentration of total nitrogen (TN) (between 40 and 60 mg/L, mainly nitrate) in the biological and catalytic ozonation treated dyeing and finishing wastewater needs to be reduced before discharge. The present study investigated the feasibility of using waste iron shavings as electron donor for nitrogen removal by biological denitrification. Two anoxic sequencing batch reactors (AnSBR) were continuously operated for more than 100 days. The results showed that the TN removal efficiency increased from 12% in the control reactor (AnSBR-C) to 20% in the reactor with waste iron shavings (AnSBR-Fe). The TN removal was mainly achieved by the reduction of nitrate by heterotrophic denitrification and autotrophic denitrification for AnSBR-Fe. The residual COD (38.4 mg/L) in the effluent of AnSBR-Fe was higher than that (22 mg/L) in the effluent of AnSBR-C, which could be due to that the bacteria preferred to use iron instead of the recalcitrant organics that present in the wastewater. Furthermore, 3DEEM, UHPLC-QTOF and GC–MS analysis were used to characterize the organics in the wastewater, and the results showed that the addition of waste iron shavings affected the degradation of organics during the biological denitrification process.

## Introduction

The wastewater from dyeing and finishing process in the textile industry is characterized by a high content of organic pollutants, which poses a serious environmental pollution if it is not properly treated (Lu et al. 2010; Wu et al. 2016). The removal of organic pollutants in the dying and finishing wastewater (DFW) has been well studied previously (Hai et al. 2007; Pang and Abdullah 2013; Sarayu and Sandhya 2012), and the typically used process for organic removal in textile industry is hydrolysis and acidification, aeration tank and secondary sedimentation. The typically used process can also be followed by advanced oxidation to further reduce the organic content in the wastewater (Wu et al. 2016). The effluent chemical oxygen demand (COD) can meet the latest discharge limitation for dyeing and finishing in the textile industry by the above process. However, the problem still remains since the effluent TN (TN) (generally >40 mg/L) is higher than the discharge limitation (20 mg/L, GB 4287-2012 for dyeing and finishing in the textile industry), which has not been attracting much attention. For instance, a new catalytic ozonation process was applied in a textile plant in pilot scale to remove the organic pollutants from bio-treated DFW based on our previous study (Wu et al. 2016), and the TN after the advanced oxidation was ranged from 46 to 58 mg/L. The TN is mainly composed of nitrate (>90% of the TN), and therefore the removal of nitrate from such wastewater remains to be solved.

Nitrate can be removed by physical–chemical treatment processes such as ion exchange and reverse osmosis, which are relatively expensive to operate (Richards et al. 2010; Samatya et al. 2006; Shin and Cha 2008). Biological denitrification is a well developed and widely applied process, which generally has low operational cost (An et al. 2009). However, biological denitrification requires electron donor for nitrogen removal. Although there are still organics in the effluent of treated DFW, which can be used as electron donor, they generally recalcitrate against biodegradation. Therefore, additional electron donor has to be added in order to achieve good performance of nitrate removal. The electron donor can be both organic compounds (e.g. acetate, methanol et al.) and inorganic compounds (e.g. H_2_, iron et al.) (An et al. 2009). The use of organic compounds for biological denitrification by heterotrophic bacteria can produce excessive biomass and soluble microbial products, which requires further treatment (Mansell and Schroeder 2002), while the use of inorganic compounds can avoid this problem (Schnobrich et al. 2007). Although both H_2_ and nano zero-valent iron (NZVI) have been studied as electron donor for denitrification (Chen et al. 2014; Shin and Cha 2008; Xia et al. 2010), they are generally limited by the relatively high cost and technical difficulties.

Waste iron shavings has relatively low cost and is abundant in China, and therefore can be used as a cheap electron donor for denitrification (Ma and Zhang 2008). Waste iron shavings has been previously used for enhanced biological treatment of industrial wastewater to remove chlorinated aliphatic compounds, organic dyes, nitrobenzenes and chlorinated phenols et al. (Agrawal and Tratnyek 1996; Gillham and O’Hannesin 1994; Ma and Zhang 2008; Yin et al. 2012). However, it has not been applied for biological denitrification. The process performance of biological denitrification based on waste iron shavings remains to be investigated. In addition, most of the previous studies focusing on the nitrate removal by external inorganic electron donor were based on synthetic wastewater (An et al. 2009; Chen et al. 2014; Shin and Cha 2008), and how the addition of such external electron donor affected the transformation of organics that present in the wastewater is not known. Especially for the present study, where the biological and catalytic ozonation treated DFW was used, there were refractory organics in the wastewater. The degradability and transformation of such refractory organics in the waste iron shavings based biological denitrification process needs to be elucidated.

Based on the above considerations, the present study aimed to investigate the feasibility of using waste iron shavings for nitrogen removal from the biological and catalytic ozonation treated DFW by biological denitrification. A comparative analysis of biological denitrification with and without waste iron shavings was conducted. The removal efficiency of nitrate, the changes of nitrogen species, and the degradation and transformation of organics were evaluated.

## Materials and methods

### Wastewater, inoculum and waste iron shavings

**Fig. 1:**
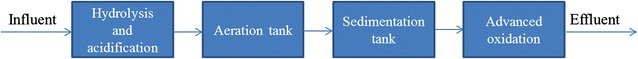
The typical process for the treatment of dyeing and finishing wastewater

The wastewater used in the present study was the effluent of a full scale wastewater treatment plant located in a typical dyeing and finishing industry cluster in southeast China (Wu et al. 2016), and the DFW treatment process in the factory is shown in Fig. 1. The concentrations of TN, nitrate, nitrite, ammonia and COD were in the range of 46.8–58.7, 45.4–56.8, 0.9–3.7, 0.02–0.15 and 42–68 mg/L, respectively. The inoculum used for biological denitrification was the activated sludge obtained from Quyang wastewater treatment plant in Shanghai, and the mixed liquor volatile suspended solids (MLVSS) and the mixed liquor suspended solids MLSS were 2100 and 2800 mg/L. Waste iron shavings was collected from a metal machinery plant using 38CrMoAl steel and washed by commercial detergent to remove surface pollutants, especial oil stain.

### Experimental set-up

**Fig. 2:**
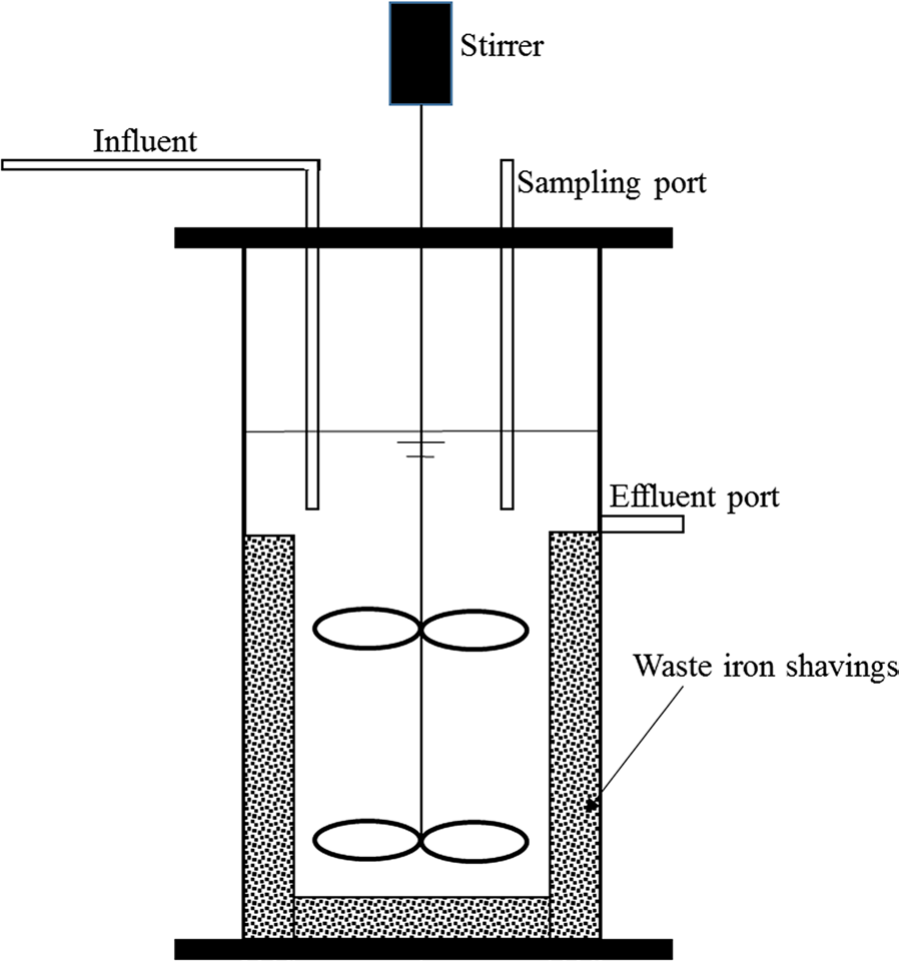
Reactor configuration

Two 6 L reactors with working volume of 4.8 L were used as the anoxic sequencing batch reactors (AnSBR) for biological denitrification (Fig. 2). One reactor (AnSBR-Fe) was filled with waste iron shavings at concentration of 62.5 g/L, and the other one (AnSBR-C) was used as control without the addition of waste iron shavings. The inoculum was added to the reactors with the final concentration of MLSS 2000 mg/L. Both reactors were mixed by mechanical stirrers at 120 rpm. The HRT of the two reactors were controlled at 32 h. The AnSBR cycle was 360 min in total, with 5 min feeding, 240 min reaction, 110 min settling, and 5 min drainage.

Batch experiments were also conducted to investigate the nitrogen removal pathways in AnSBR-Fe. Three batch experiments were carried out. Batch experiment I was used to test the nitrogen removal by the organics in the wastewater and waste iron shavings. Mixture of 200 mL was obtained from AnSBR-Fe, and centrifuged. The centrifuged sludge and 200 mL wastewater were added to 500 mL serum bottles. Waste iron shavings were also added with the final concentration of 62.5 g/L. The bottles were flushed with nitrogen and then incubated in a shaker with 120 rpm. Batch experiment II was used to test the nitrogen removal without waste iron shavings. The difference from batch experiment I was that waste iron shavings were not added. Batch experiment III was used to test the nitrogen removal by waste iron shavings without biological functions. The difference from batch experiment I was that the centrifuged sludge was not added. Each experiment was conducted in triplicates.

### Analytical methods

Samples from the two continuous AnSBRs were taken periodically for routine analysis. COD, MLSS, MLVSS, nitrate, nitrite, ammonia, TN, total iron, Fe^3+^ and Fe^2+^ were measured according to APHA (1995). The TOC and TN were determined with a TOC analyser (Shimadzu TOC-L, Japan).

During steady-states, samples from the two reactors were also collected for the characterization of the residual organics. The 3DEEM was determined using a fluorometer (HORIBA Jobin–Yvon FluoroMax-4, France). The organic species were analyzed using GC–MS (Shimadzu GCMS-QP2010 SE, Japan with a HP5-MS column) and UHPLC-QTOF (Agilent 1290 UHPLC, Agilent 6540 QTOF, USA with Agilent ZORBAX SB-C18 HD column). Detailed information about the analysis can be found in our previous study (Wu et al. 2016). The steady-states were considered to be achieved when the nitrate removal was stable for at least 2 weeks.

The theoretical COD amounts required for the removal of nitrate and nitrite were calculated based on the values of 2.86 and 1.71 gCOD/gNO_2_-N respectively (Medigue and Eddy 2002).

## Results

### Performances of the reactors

**Fig. 3:**
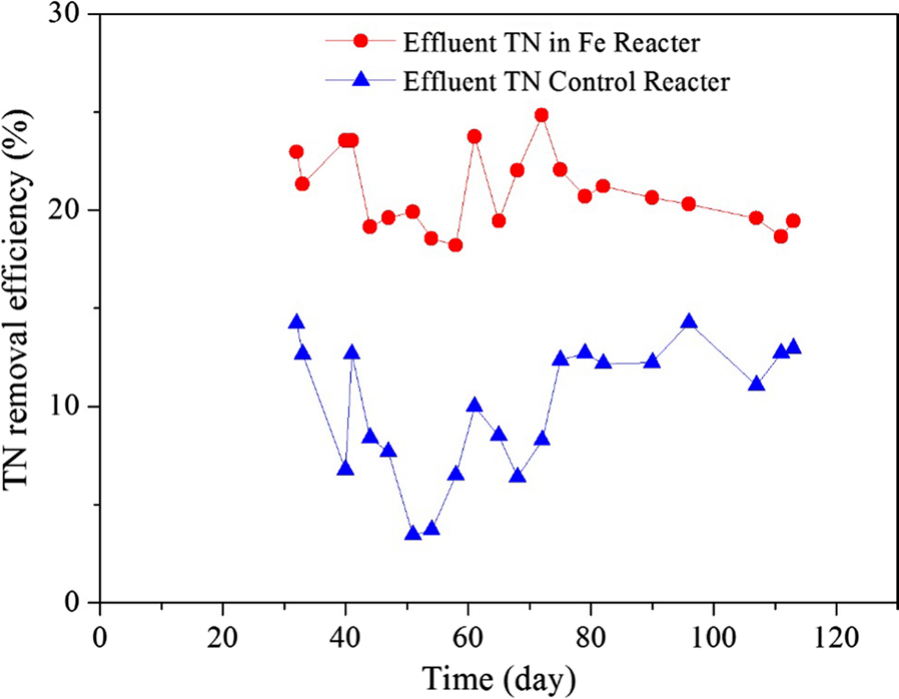
TN removal efficiency

**Fig. 4:**
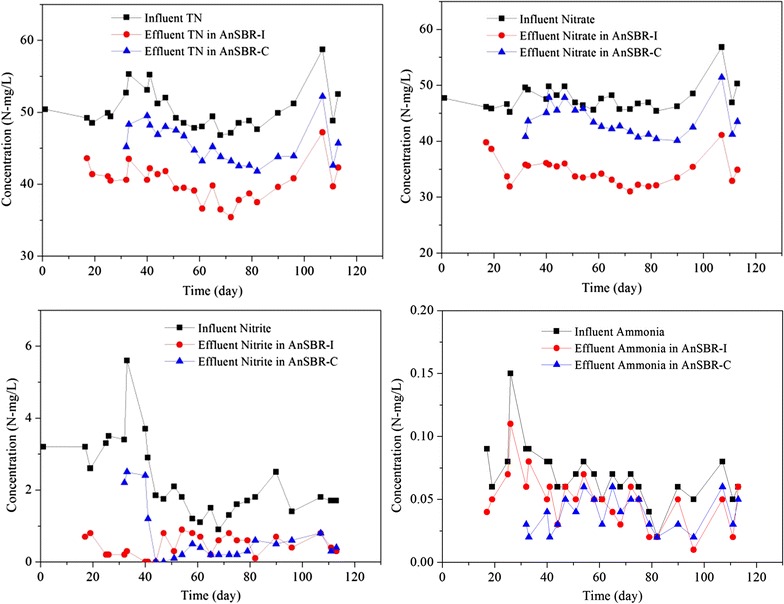
The concentrations of TN, nitrate, nitrite, and ammonia in the influent and effluent

**Fig. 5:**
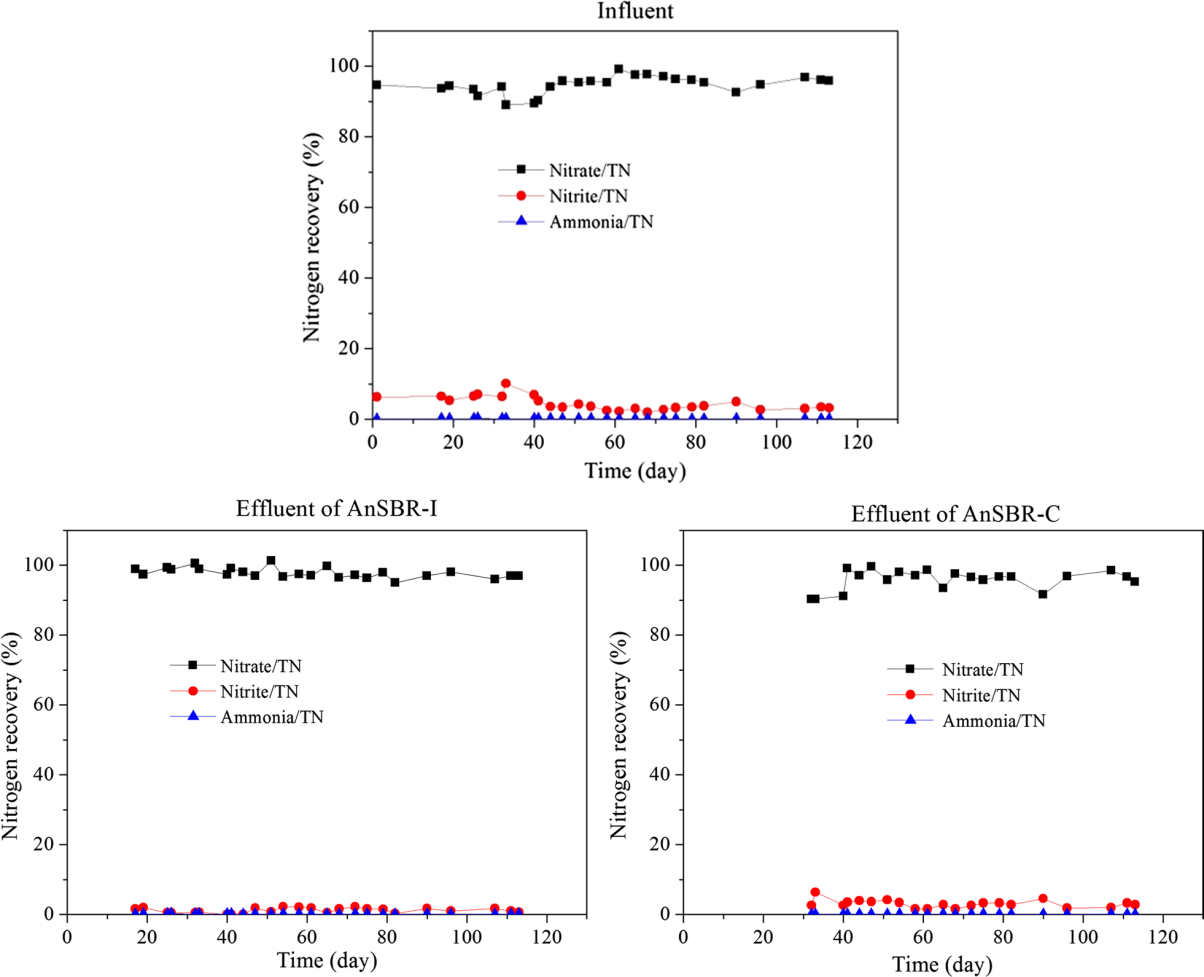
Nitrogen mass balance

The two AnSBR were operated for more than 100 days until relatively stable TN removal efficiencies were achieved. As shown in Fig. 3, TN removal efficiencies were around 20% of AnSBR-Fe, which was higher than that (12%) of AnSBR-C. The concentrations of TN, nitrate, nitrite and ammonia in the influent and effluent of both reactors are shown in Fig. 4. The changes of TN were consistent with the changes of nitrate, and it could be due to that TN was mainly composed by nitrate. Both the concentrations of nitrite (<5 mg/L) and ammonia (<1 mg/L) were in very low levels. Figure 5 shows the nitrogen mass balances for the influent and effluent. It is obvious nitrate was the main component (>90%) in both influent and effluent.

The COD concentration was also measured and it is shown in Table 1. The decrease of COD was found in both AnSBRs, which also indicated parts of the organics in the wastewater were removed. It should be noted that the effluent COD of AnSBR-C was lower than that of AnSBR-Fe. Fe^2+^ and Fe^3+^ would be produced if iron was used for denitrification (Shin and Cha 2008), however, the concentration of soluble Fe ion in the effluent of AnSBR-Fe (4 mg/L) was even lower than that (5.4 mg/L) in the influent. The concentrations of MLSS and MLVSS are also shown in Table 1. The concentration of MLSS in AnSBR-Fe was 3733 mg/L, which was 88% higher than that (1981 mg/L) of AnSBR-C, and the concentration of MLVSS in AnSBR-Fe (884 mg/L) was 23% higher than that (718 mg/L) in AnSBR-C.

**Table 1:** Summary of the selected parameters during steady-states of the reactors

	Influent	AnSBR-I	AnSBR-C
COD (mg/L)	52.3 ± 3.1	38.4 ± 1.5	22 ± 1
The ratio of consumed COD to calculated COD required for denitrification	/	0.46 ± 0.02	1.7 ± 0.2
MLSS (mg/L)	/	3733 ± 73	1981 ± 325
MLVSS (mg/L)	/	884 ± 0.7	718 ± 86

**Fig. 6:**
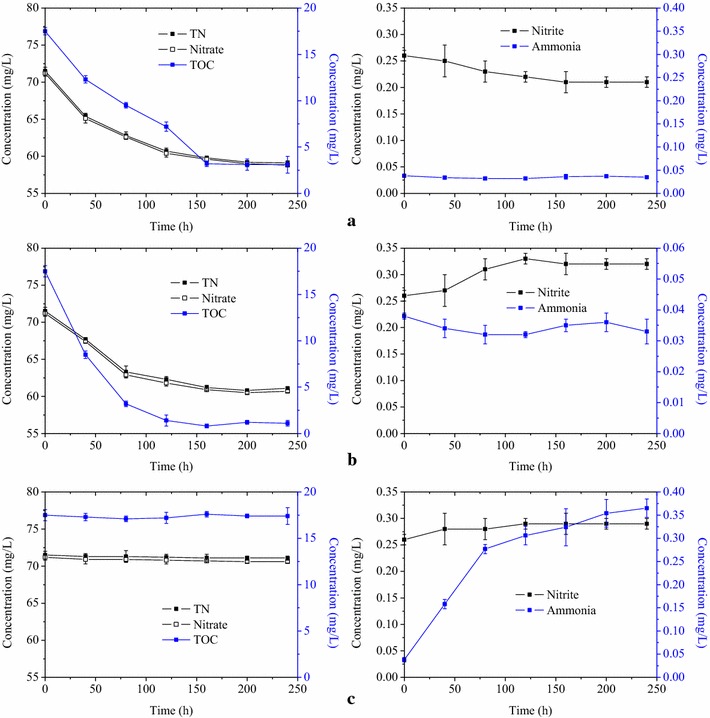
The time courses of TN, nitrate, TOC, nitrite and ammonia in batch experiments. **a** Experiment with waste iron shavings and sludge, **b** experiment with only sludge, **c** experiment with only waste iron shavings

The results from batch experiments are shown in Fig. 6. Figure 6a showed that around 17% of the TN in the wastewater was removed when both sludge and waste iron shavings were present. The decrease of TOC was also observed, further indicating that the nitrogen was removed via biological denitrification by heterotrophic bacteria. Figure 6b showed that the TN removal efficiency was around 14% when waste iron shavings were absent, which indicated that the presence of waste iron shavings improved the TN removal efficiency by 3% in the batch experiments. Figure 6c showed that the removal of TN by waste iron shavings via chemical reaction could be ignored in the present study. The above results indicated that the TN removal with the addition of waste iron shavings to AnSBR was mainly achieved by heterotrophic and autotrophic denitrification.

### Characterization of the dissolved organics

**Fig. 7:**
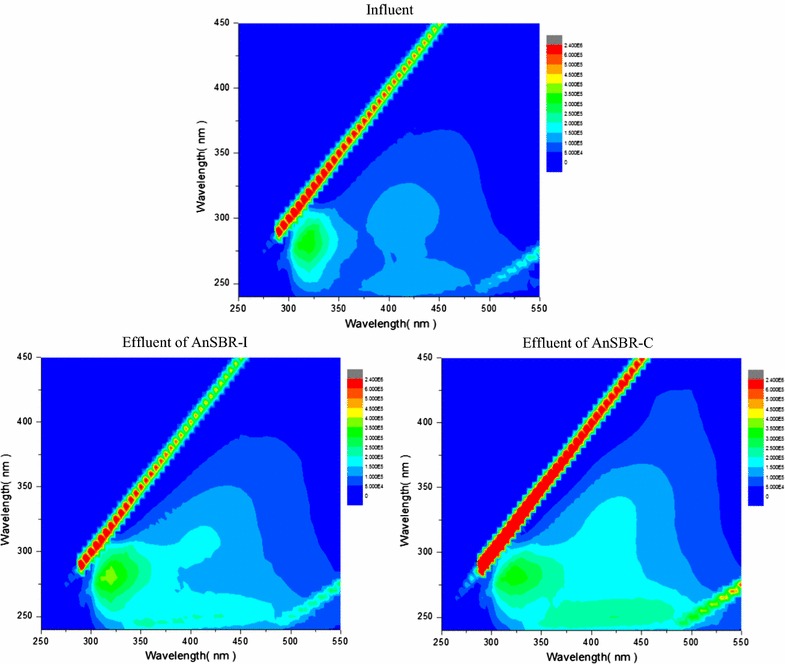
3DEEM of different samples

Figure 7 shows the 3DEEM fluorescence of the wastewater before and after treatment. There was only one peak for the raw wastewater, which corresponded to protein like compounds (Em/Ex = 275/325) (Chen et al. 2003; Wu et al. 2016). After treatment, the peak was still present in the samples obtained from both reactors, which indicated protein like compounds were not degraded. A peak at Em/Ex = 250/425, representing fulvic acid like compounds, appeared after treatment. UHPLC-QTOF was used to detect the changes of organic pollutant species. A total of 574 species were detected in the raw wastewater, and most of the detected species (92%) were strong polar species (Table 2). The effluent of AnSBR-C contained 580 species, while the number of detected species was only 547 for the effluent of AnSBR-Fe. The main organic species detected by GC–MS are shown in Table 3. The total peak area of main pollutants in raw wastewater (6080649) was higher than that in AnSBR-C (3180570) and AnSBR-Fe (3904995), which was consistent with the changes of COD concentrations. About half of the organic species in raw wastewater were reduced in different extents in both AnSBR-C and AnSBR-Fe.

**Table 2:** Summary of the results from UHPLC-QTOF

	Influent	AnSBR-I	AnSBR-C
Medium or weak polar species^a^	44 (8%)^b^	43 (8%)	54 (9%)
Strong polar species^a^	530 (92%)	504 (92%)	526 (91%)
Total species	574	547	580

^a^The species with retention time ≤2 min were regarded as strong polar species, while the species with retention time 2 min were regarded as medium or weak polar species

^b^The number in the bracket was the ratio of the species to the total species

**Table 3:** Summary of the results from GC–MS

Name of the compounds	Influent		AnSBR-I		AnSBR-C	
	PA^a^	RPA^b^	PA	RPA	PA	RPA
6,6’-Dimethyl-5,5’,8,8′-tetramethoxy-2,2′-binaphthylidene-1,1′-dione	196,235	3.09	220,130	5.6	201,486	5.85
Acetic acid, ethyl ester	279,478	4.41	308,757	7.86	355,725	10.34
Dodecane	196,961	3.11	140,111	3.57	155,585	4.52
1-Dodecanamine,* N*,*N*-dimethyl-	2,761,717	43.56	1,148,843	29.25	682179	19.82
Phenol, 2,4-bis(1,1-dimethylethyl)	22,307	0.35	139,736	3.56	98,437	2.86
2-Propanone	519,281	8.19	282,789	7.2	157,243	4.57
1,2-Benzenedicarboxylic acid, bis(2-methylpropyl) ester	223,301	3.52	87,331	2.22	289,873	8.42
n-Hexadecanoic acid	260,061	4.1	358,363	9.12	104,911	3.05
6-Methylfuro[2,3-c]pyrid-5-one	156,178	2.46	102,932	2.62	147,336	4.28
2-(2-N-Benzyl-N-methylaminoethyl)-4,5-dimethoxyphenylaceticacid,methyl ester	372,461	5.87	131,375	3.34	78,300	2.28
Docosanoic acid	82,071	1.29	135,536	3.45	85,055	2.47
Acetic acid, decyl ester	20,003	0.32	ND	ND	28,224	0.82
Sulfurous acid, 2-propyl undecyl ester	49,278	0.78	49,298	1.25	33,104	0.96
*N*-Methyl-*N*-benzyltetradecanamine	68,133	1.07	ND	ND	ND	ND
Nonane, 4,5-dimethyl-	50,038	0.79	39,742	1.01	36,710	1.07
1-Phenanthrenecarboxylic acid	ND	ND	90,896	2.31	240,252	6.98
3,6-Dioxa-2,7-disilaoctane, 2,2,4,7,7-pentamethyl-	ND	ND	21,995	0.56	40,817	1.19
Octadecane (CAS)* n*-Octadecane	70,196	1.11	52,349	1.33	31,604	0.92
1,1-Dibromo-2-(2,2-dimethylpropyl)cyclopropane	85,848	1.35	74,477	1.9	ND	ND
Tetrasiloxane, decamethyl-	67,302	1.06	57,640	1.47	30,393	0.88
1,2-Benzenedicarboxylic acid, bis(2-ethylhexyl) ester	101,833	1.61	73,792	1.88	65,536	1.9
2-Bromo dodecane	49,778	0.78	37,346	0.95	27,326	0.79
Promecarb 2,4-dinitrophenylether	ND	ND	61,276	1.56	ND	ND
3-(2-Methoxymethoxy-ethylidene)-2,2-Dimethyl-Bicyclo[2.2.1]Heptane	ND	ND	7982	0.2	ND	ND
2-Bromotetradecane	41,372	0.65	67,606	1.72	70,099	2.04
2,5-Dimethyl-4-methoxyphenol	75,184	1.19	40,286	1.03	78,727	2.29
Eicosamethylcyclodecasiloxane	115,403	1.82	48,971	1.25	40,080	1.16
Silikonfett SE30(GREVELS)	95,459	1.51	58,809	1.5	51,194	1.49
1H-Purin-6-amine, [(2-fluorophenyl)methyl]-	120,771	1.9	66,627	1.7	50,374	1.46

Not detected

^a^Peak area

^b^Relative peak area (%)

## Discussion

The results from the present study showed that the addition of waste iron shavings increased the nitrogen removal efficiency from the biological and catalytic ozonation treated dyeing and finishing wastewater. For AnSBR-C, the removal of nitrogen could be related with the organics in the wastewater, which was thought to be refractory organics since they have undergone biological and chemical oxidation. However, it seems that this part of organics could still be utilized by denitrifying bacteria. The addition of waste iron shavings provided additional electrons and therefore obviously increased the TN removal efficiency. The low ammonia concentration in the effluent indicated that nitrate was mainly removed by biological denitrification (Shin and Cha 2008), but not by the abiotic reactions as shown in Eq. 1 (Suzuki et al. 2012), further showing the advantage of biological denitrification based on iron.

1 $$ {\text{NO}}_{ 3}^{ - } + 3 {\text{Fe}}^{0} + {\text{H}}_{ 2} {\text{O}} + 2 {\text{H}}^{ + } \to {\text{NH}}_{ 4}^{ + } + {\text{Fe}}_{ 3} {\text{O}}_{ 4} $$

The effluent COD of AnSBR-C was lower than that of AnSBR-Fe. The reason could be that the bacteria preferred to use iron instead of the refractory organics in the wastewater. In AnSBR-C, the bacteria had to use the refractory organics, and therefore less COD was left in AnSBR-C compared to AnSBR-Fe. The ratio of removed COD to the theoretical COD needed for nitrate and nitrite removal was calculated and is shown in Table 1. The ratio in AnSBR-C was higher than 1, and there were two possible reasons. First, part of the COD from suspended solid in the wastewater might be absorbed by the sludge. Second, the growth of microorganisms might also consume part of the COD. The above mentioned COD were not used for denitrification although it was accounted for the total removed COD, which thereby resulted in the ratio higher than 1. However, the ratio in AnSBR-Fe was 0.46, which means the electrons provided by the removed COD was not enough for denitrification, and therefore the additional electrons should be mainly derived from the waste iron shavings. The lower concentration of soluble Fe ion in the effluent of AnSBR-Fe (4 mg/L) compared to that in the influent could be due to that both ferrous and ferric salts were good flocculant which precipitated easily in neutral pH (Rodrigues et al. 2013; Wang et al. 2008; Zhao et al. 2011). The ratio of MLVSS/MLSS was only 0.24 in AnSBR-Fe, while it was about 0.36 in AnSBR-C, and the result indicated that more inorganic compounds were present in the MLSS of AnSBR-Fe, which could be related with the precipitation of ferric and ferrous salts. The concentration of MLVSS represents the concentration of microorganisms in the system, and it should be noted that MLVSS in AnSBR-Fe did not include all the microorganisms since some microorganisms could be attached to the waste iron shavings and therefore not be quantified by the measurement of MLVSS.

In the batch experiments, the presence of waste iron shavings improved the TN removal efficiency by 3% (the TN removal efficiencies increased from 14 to 17%) in the batch experiments (Fig. 6b), while the TN removal efficiency was improved by 8% (the TN removal efficiencies increased from 12 to 20%) with the addition of waste iron shavings in the continuous experiments (Fig. 3). The reason might be that the microorganisms utilizing waste iron shavings were mostly attached in the surface of waste iron shavings, while the sludge used for the batch experiment was obtained from the liquid phase. The observation of biofilm formation during iron corrosion was also reported before (Lee and Characklis 1993). As previously mentioned, the TN removal by heterotrophic denitrification should be lower than 12% (the value in AnSBR-C) in AnSBR-Fe since the effluent COD of AnSBR-Fe was higher than that of AnSBR-C. Since the TN removal by waste iron shavings via chemical reaction could be negligible (Fig. 6c), the TN removal efficiency by autotrophic denitrification should be higher than 8% in AnSBR-Fe considering the total TN removal efficiency of 20%.

3DEEM fluorescence analysis shows that protein like compounds were not degraded in both AnSBR-Fe and AnSBR-C. By comparing the 3DEEM fluorescence of the effluent from the two reactors (Fig. 7), it was obvious that the intensity of fulvic acid-like peak in the sample obtained from AnSBR-C was higher than that of AnSBR-Fe, which could be due to that more organics were degraded in AnSBR-C as shown in Table 1 and therefore more fulvic acid-like compounds were formed.

The effluent of AnSBR-C contained 580 species by UHPLC-QTOF analysis, which was slightly higher than that in the raw wastewater, and it could be related with the formation of new species in the reactor. For the effluent of AnSBR-Fe, the number of detected species was only 547, which was lower than that in both raw wastewater and effluent from AnSBR-C. The above results clearly showed that the organic transformation in AnSBR-C and AnSBR-Fe were different. It should be noted that decreased organic species in the effluent of AnSBR with waste iron shavings does not necessarily mean the low organic concentration in the effluent as can be seen from the COD results (Table 1).

GC–MS analysis showed that 1-Dodecanamine, N,N-dimethyl- was dominant in all the three samples, which was also detected in the wastewater in our previous study (Wu et al. 2016). A better degradation efficiency was observed in AnSBR-C compared to AnSBR-Fe, which might be related with the higher degradation of COD in AnSBR-C. Similar results were also found for other organic species including 2-Propanone, 2-(2-N-Benzyl-N-methylaminoethyl)-4,5-dimethoxyphenylaceticacid, methyl ester, Octadecane etc. The above results clearly showed that the degradation efficiency of some organic species might be reduced due to the addition of waste iron shavings. The rest organic species were not degraded or even enriched. It could be due to that these organics species were recalcitrant to biological degradation or produced by the transformation of other organic compounds.

The results from the present study clearly showed that waste iron shavings could be used to improve the nitrogen removal efficiency from the biological and catalytic ozonation treated dyeing and finishing wastewater, where the residual organics were not enough to be utilized for biological denitrification. The nitrogen removal efficiency was around 20% in AnSBR-Fe, and the corresponding TN in the effluent were in the range 35–45 mg/L, which was still higher than the discharge requirement (<20 mg/L). In order to further increase the TN removal efficiency, pretreatment of the waste iron shavings to increase the surface area may be a solution, which would increase the contact between waste iron shavings and microorganisms and therefore increase the electron transfer rate. For instance, NZVI was used as electron donor for biological denitrification, which resulted in high nitrogen removal efficiency since it has huge surface area (An et al. 2009; Shin and Cha 2008). However, NZVI was not feasible for wastewater treatment in practice due to its relatively high cost, difficulty to be stored and easiness to be oxidized. Therefore, the increase of the surface area of waste iron shavings would be a cost-effective method for biological denitrification. Another way to further increase the TN removal efficiency would be the addition of organic carbon sources (e.g. acetate, methanol) in order to meet the discharge requirement.

## Abbreviations

TN: total nitrogen
AnSBR: anoxic sequencing batch reactors
DFW: dying and finishing wastewater
COD: chemical oxygen demand
NZVI: nano zero-valent iron
